# The plant matrix of *Artemisia annua* L. for the treatment of malaria: Pharmacodynamic and pharmacokinetic studies

**DOI:** 10.1371/journal.pone.0322835

**Published:** 2025-05-07

**Authors:** Fujie Xu, Xiaohang Shan, Jialin Li, Jing Li, Jiqiao Yuan, Daozeng Zou, Manyuan Wang

**Affiliations:** 1 Beijing Key Laboratory of TCM Collateral Disease Theory Research, School of Traditional Chinese Medicine, Capital Medical University, Beijing, China; 2 Xiyuan Hospital, China Academy of Chinese Medical Sciences, Beijing, China; Waterford Institute of Technology, IRELAND

## Abstract

Artemisinin-based combination therapies (ACTs) constitute the principal strategy for combating malaria in contemporary times, and research into the multifaceted components of *Artemisia annua* L. (*A. annua*) has garnered widespread interest among scientists. The aim of this study was to prepare *A. annua* extracts (nACTs) and to explore whether nACTs have higher bioavailability and efficacy than artemisinin (ART) alone due to its multiple bioactive components. Initially, the *in vivo* antimalarial activity of nACTs was evaluated by two murine malaria models. The results revealed that the antimalarial effect of nACTs was about 10-fold higher than that of ART alone when administered at the same dosage of ART. Then, we analyzed the pharmacokinetic characteristics of nACTs in rat plasma. Remarkably, nACTs exhibited significantly enhanced oral bioavailability, longer half-life as well as extended mean retention time in rats. In addition, the impact of nACTs on P-glycoprotein (P-gp) was evaluated using the Caco-2 cell line. The results showed that both ART and nACTs reduced the efflux rate of the P-gp substrate rhodamine 123 (R123) and induced the expression of P-gp in Caco-2 cells over a range of concentrations. nACTs had certain components-deoxyartemisinin (DEART), artemisinic acid (AA), and dihydroartemisinic acid (DHAA)-that inhibited the efflux and translocation of P-gp and facilitated the reduction of ART efflux. In conclusion, *A. annua* extracts significantly improved the antimalarial efficacy and bioavailability compared with ART.

## Introduction

Due to the 2019 coronavirus disease (COVID-19) pandemic, malaria control services have been disrupted, and the number of malaria cases and deaths has increased significantly [1]. According to the latest report from the World Health Organization (WHO), it is estimated that in 2023, there were 263 million cases of malaria and 597,000 malaria-related deaths globally, an increase of approximately 11 million cases compared to 2022 [2]. Artemisinin (ART) is an antimalarial active ingredient discovered in *Artemisia annua* L. (*A. annua*) by Youyou Tu’s research team in 1972. However, ART has the disadvantages of poor solubility, low bioavailability, and a high recrudescence rate of malaria. Therefore, it is generally available for clinical application in the form of ART derivatives (dihydroartemisinin, artesunate, artemether, etc.), which have cured numerous malaria patients worldwide [3,4]. The introduction of artemisinin-based combination therapies (ACTs), recommended by WHO in 2001 as the first-line treatment for malaria, provided a much-needed and highly effective antimalarial treatment, and it became the first-line treatment for uncomplicated *Plasmodium falciparum* (*P. falciparum*) malaria in all endemic countries [5,6]. Nevertheless, the emergence of partial resistance to ART in the Greater Mekong Subregion (GMS) and the accompanying high ACT treatment failure rates due to partner drug resistance mean that new regimens and strategies using existing antimalarials will be required before new compounds can be deployed [7–9].

In recent years, studies have found that dried leaves of *A. annua* are more efficacious than ART alone in clearing malaria from the bloodstream and are more bioavailable [10–12]. Whole plant preparations have also been reported in the literature to be effective in treating patients with artesunate-resistant malaria [13]. In particular, flavonoids enhance the antimalarial activity of ART [14,15]. These results suggest that the complex matrix of chemicals in *A. annua* may work in conjunction with ART, and whole plant preparations have been suggested as an alternative to ACTs [11]. At the same time, the WHO has advocated for increased research on the antimalarial properties of the *A. annua* plant [16]. Our group previously used a four-component combination of ART, artemisinin B, artemisinic acid, and scopoletin (1:1:1:1) to increase antimalarial efficacy by nearly four-fold compared to pure ART [17]. In our recent study it was found that *A. annua* extracts not containing ART still have antimalarial activity, which opens up the possibility of exploring new ACTs [18].

P-glycoprotein (P-gp; MDR1 gene product) is an ABC transporter protein that is ubiquitously distributed in a variety of human tissues, such as the brain, kidney, liver, and intestinal cells [19,20]. P-gp acts as an efflux pump that plays an important role in drug absorption and distribution, transporting a variety of compounds from the intracellular to back to the extracellular space, thereby decreasing intracellular drug concentrations or altering pharmacokinetics [21,22]. Furthermore, P-gp substrates can bind specifically to P-gp and translocate P-gp out of the cell, while this efflux can be inhibited by P-gp inhibitors [23–25]. Some natural compounds such as flavonoids, alkaloids, terpenoids, and saponins can interfere with the transcriptional expression of P-gp and/or directly inhibit its function, thus further contributing to the reduction of drug efflux [26,27]. Here we inherited the idea of ‘ether-neutral-dry’ active site by relying on the high-quality *A. annua* resources in China. Petroleum ether, a low-polarity solvent, was used to extract and purify *A. annua* to obtain low-cost, dose-controlled natural antimalarial drugs, *A. annua* extracts (nACTs). Five representative components of the extract were quantified simultaneously using Ultra Performance Liquid Chromatography (UPLC). The antimalarial effects of nACTs were evaluated by two malaria models and *in vivo* pharmacokinetic interaction studies were performed using rats. The herbal interactions of nACTs with P-gp were investigated by an *in vitro* Caco-2 cell model. The synergistic antimalarial effect of *A. annua* natural multi-components with ART was strongly demonstrated.

## Materials and methods

### Materials and reagents

*A. annua* extracts (natural ACTs, nACTs, the original medicinal materials of *A. annua* were harvested from Yongzhou, Hunan, batch number: 20201202, identified by Professor Wang Manyuan, School of Traditional Chinese Medicine, Capital Medical University as the leaves and stems of *A. annua* L. cultivar). ART (purities > 98.8%, batch number: 100202–202005) was purchased from the China National Institute for Food and Drug Control. Giemsa stain, Rhodamine123 (R123), and heparin sodium (titer ≥ 140 U/mg) were purchased from Beijing Solarbio Science Technology Co., Ltd. (Beijing, China). Soybean oil purchased from Aladdin. Verapamil was purchased from Sigma-Aldrich. Dulbecco’s modified eagle’s medium (DMEM, high-glucose), fetal bovine serum, nonessential amino acids, 0.25% trypsin-EDTA solution, Hank’s balanced salt solution (HBSS), and antibiotic-antimycotic were purchased from Gibco BRL Life Technology (Grand Island, NY, USA). Cell counting kit-8 (CCK-8) was purchased from Dojindo Laboratories (Japan). Cell culture flasks and Transwell® polycarbonate inserts (12 mm diameter, 0.4 μm pore size) were obtained from Corning Costar Corp. (Bedford, MA, USA). P-glycoprotein rabbit monoclonal antibody was purchased from Abcam. internal standard (IS) Buspirone hydrochloride (purity 99.9%) was purchased from TRC, Canada, dimethyl sulfoxide (DMSO, Amresco, USA), and purified water (Watsons Trademark Co., Ltd.). Acetonitrile (HPLC grade) and Formic acid (HPLC grade) were purchased from Fisher (Massachusetts, USA). Other chemicals were analytical reagent grade.

### Animals

ICR mice (female, 6 weeks, 18 ~ 22 g) and Sprague Dawley rats (male, 6 ~ 8 weeks old, 280 ~ 320 g) were obtained from Beijing Vital River Experimental Animal Technical Co., Ltd. (Beijing, China). All animals were allowed free access to food and water for one week prior to use in an SPF-grade laboratory, maintained at a temperature of 22–26°C, a humidity of 50–60%, and a 12/12 h light-dark cycle. This study was carried out in strict accordance with the recommendations in the Guide for the Care and Use of Laboratory Animals of the National Institutes of Health. The protocol was approved by the Committee on the Ethics of Animal Experiments of Capital Medical University (Protocol Number: AEEI-2019–145). In the experiment, animals were anesthetized with 1% sodium pentobarbital at an intraperitoneal dose of 40 mg/kg. All animals were euthanized by carbon dioxide inhalation at the end of the experiment.

### Preparation of the *A. annua* extracts (nACTs) and isolation of major components

The original herbal material of *A.* annua was extracted by reflux with 10 times the amount of petroleum ether (60°C ~ 90°C) 4 times, each time for 1 h. The filtrates were combined and purified by column purification with silica gel (5:4 g/g original herb to silica gel by volume). The elution was then carried out with 20% ethyl acetate-petroleum ether solution, and the elution was considered to be complete when no ART spots appeared in the eluate by thin-layer plate detection. The eluates were combined, concentrated and dried.

Arteannuin I (ARTI), Deoxyartemisinin (DEART), Dihydroartemisinic acid (DHAA), and Artemisinic acid (AA), obtained by isolation and preparation from *A. annua* extracts, were identified by high-resolution mass spectrometry (HRMS, Waters Xevo G2-S, Waters, USA) and nuclear magnetic resonance apparatus (NMR, Bruker 500 MHz AVANCE II NMR Spectrometer). The validated UPLC method [28] was employed for the quantification of five sesquiterpene constituents (ART, DEART, AA, DHAA, and ARTI) in nACTs.

### *In vivo* antimalarial efficacy

*In vivo* evaluation of antimalarial activity was performed using the classical Peters’ four-day inhibition method [17,29,30]. A murine malaria model was constructed by intraperitoneal injection of 5.00 × 10^7^ *Plasmodium yoelii* (*P. yoelii*)- or *Plasmodium berghei* (*P. berghei*)-infected erythrocytes. Malaria mice were randomized into 7 groups (n = 5) including the control group (given equal doses of medicinal soybean oil), ART alone group (5.85, 11.69, 23.38 mg/kg), and nACTs group (20.00, 40.00, 80.00 mg/kg). Mice were gavaged once 4 hours after infection with medicated soybean oil or the test drug (gavage standard was 0.20 mL/10 g), and the drug was administered orally for 4 consecutive days. Four hours after the last dose, blood was collected from the tail tip of the mice to make a thin blood smear. The smear was air-dried, fixed in methanol, stained with Giemsa for 30 minutes, then rinsed with distilled water, air-dried at room temperature, and visualized for malaria infection under a microscope (OLYMPUS BX43) at 100x oil immersion. The inhibition ratio (%) for each group was calculated using the following equations:


$$Inhibitionratio\left(\%\right)=\left(infectionratio_{controlgroup}-infectionratio_{treatmentgroup}\right)/infectionratio_{controlgroup}\;\times \;100$$

### Pharmacokinetic studies in rats

Twelve male rats were randomly divided into two groups after 12 hours of fasting and were orally administered 100 mg/kg ART or 352 mg/kg nACTs, respectively. At 0.08, 0.25, 0.5, 1, 1.5, 2, 3, 4, 8, 12, and 24 h after drug administration, approximately 200 μL of blood was withdrawn through the fundus venosus plexus, and placed into a centrifuge tube containing sodium heparin. Blood samples were centrifuged at 3500 rpm for 5 min at 4°C. All plasma samples were then stored at -80°C until analysis.

### Biological sample analysis

#### Solution preparation.

The five standard compounds, ART, DEART, DHAA, AA and ARTI, and the internal standard (IS) buspirone hydrochloride were dissolved in dimethyl sulfoxide to form a standard stock solution at a concentration of 1.00 mg/mL. A series of standard solutions with different concentrations were prepared by diluting the five standard stock solutions with methanol, and the final concentrations of ART, DEART, DHAA, AA and ARTI were 100, 200, 400, 800, 2000, 4000, 10000, 20000, 50000 and 100000 ng/mL, respectively. A range of standard solutions of different concentrations were prepared for calibration curves and quality control. The IS solution was diluted with acetonitrile to a final concentration of 100 ng/mL.

#### Preparation and determination of plasma samples.

All samples were stored in a refrigerator at -80°C and thawed at room temperature before processing. A 50 μL aliquot of plasma was added with 5 μL of the standard solution (or acetonitrile) and 100 μL of internal standard solution in turn, vortexed and mixed, then sonicated for 3 min, centrifuged at 14000 rpm for 10 min at low temperature. The supernatant was transferred to an autosampler vial and analyzed by UHPLC-ESI-MS/MS.

Determination of concentration changes of each major component of nACTs in plasma by LC-MS 6490 Liquid Mass Spectrometer (Agilent Technologies), Waters ACQUITY UPLC BEH C18 Column (2.1 mm × 100 mm, 1.7 μm), equipped with Waters ACQUITY UPLC C18 Van Guard Pre-column (2.1 mm × 5 mm, 1.7 μm). The mobile phase consisted of 0.1% formic acid solution (A) and acetonitrile (B). The solvent gradient was as follows: 0.01–5 min with 65%-5% B, 5–6 min with 5% B, 6–8 min with 5%-65% B, and 8–9 min with 65% B. The injection volume was 5.0 μL, the flow rate was 0.3 mL/min, and the chromatographic run time was within 9.0 min.

Five compounds, including ART, DEART, DHAA, AA, and ARTI, as well as the working standard solution of internal standard buspirone (concentration 1.00 μg/mL), were injected directly into the mass spectrometer and scanned in positive ion mode. The ESI ionization source was used and detected in MRM mode. The specific mass spectrometric conditions are shown in Table 1, the mass spectra of the daughter ions of each compound are shown in S1 Fig, and the possible cleavage patterns are shown in S2 Fig.

**Table 1:** Operating parameters of positive ion mode mass spectrometry.

Mass Spectrometry Parameters	ART	DEART	ARTI	DHAA	AA	Buspirone
Parent ion (*m/z*)	283	267	235	237	235	386
Quantitative ion (*m/z*)	247	203	161	163	217	122
Qualitative ions (*m/z*)	219	207	189	107	189	205
Quantitative ion CE (eV)	10	10	10	15	10	35
Qualitative ions CE (eV)	10	15	10	30	10	35
Gas Temp (°C)	200					
Gas Flow (L/min)	14					
Nebulize (psi)	20					
Sheath Gas Temp (°C)	250					
Sheath Gas Flow (L/min)	11					
Capillary (V)	3000					
Nozzle Voltage (V)	1500					

#### Method validation.

The method was validated according to the US Food and Drug Administration (FDA) Bioanalytical Method Validation Guidance [31] as follows: [1] specificity was assessed by analyzing blank plasma, blank plasma spiked with standard solutions and rat plasma samples after oral administration. [2] Each calibration curve was plotted using the concentration of the standard solution as the horizontal coordinate and the ratio of analyte to internal standard peak area as the vertical coordinate. The lower limit of quantification (LLOQ) refers to the lowest concentration, which is typically 10 times the signal-to-noise ratio. It has an acceptable accuracy of ± 20% and a precision of less than 20% [3]. Quality Control (QC) samples (low, medium and high concentrations) were prepared for each analyte. Five samples were analyzed at each concentration level and three consecutive batches were determined to calculate the accuracy and precision of the method. [4] Extraction recovery was assessed by the ratio of the average peak area of routinely prepared QC samples (low, medium, and high concentrations) to the post-extraction plasma sample. Likewise, matrix effect was assessed by the ratio of peak areas between post-extraction samples spiked with analytes and mobile phases spiked with the same concentration of analytes. [5] QC samples were prepared at low, medium and high concentrations, and the stability of the samples was investigated after three freeze-thaw cycles, 24 h at 4°C and 7 days at -80°C, respectively.

### Pharmacodynamics in Caco-2 cells

#### Cell culture.

The human epithelial cell line Caco-2 serves as a widely recognized model for the intestinal epithelial barrier, frequently utilized in the assessment of drug absorption and transport mechanisms [32–34]. In this study, we evaluated drug absorption and distribution *in vitro* with the help of the Caco-2 cell model. Caco-2 cells were purchased from the Chinese Academy of Medical Sciences (Beijing, China, Resource No. 3111C0001CCC000100). The cells were resuscitated and inserted into culture flasks. Caco-2 cells were passaged with DMEM high glucose medium containing 10% (V/V) FBS, 1% (V/V) non-essential amino acid solution and 100 U/mL penicillin-streptomycin double antibiotic solution, and cultured at 37°C in a 5% CO_2_ incubator. When the cells were observed in a monolayer of tight junctions with an inverted microscope and the confluence reached 80%, Caco-2 cells were digested with 0.25% trypsin-EDTA solution and inoculated into a 12-well Transwell model at a density of 1.0 × 10^5^ cells/cm^2^. The integrity of the cell monolayer was checked by measuring the transmembrane electrical resistance (TEER) of the monolayer cells with a Millicell-ERS electrode (Millipore Corp., Billerica, MA, USA). Caco-2 cells were used for transport experiments 17–21 days after inoculation [35]. Besides, only monolayers with TEER values above 400 Ω/cm^2^ were used in the study.

#### Cytotoxicity.

The cytotoxicity of nACTs, ART, DEART, DHAA, and AA was evaluated in Caco-2 cells using the CCK-8 assay. Caco-2 cells were cultured in 96-well plates for 48 h at a seeding density of 5.0 × 10^3^ cells/well before the addition of drugs. ART, DEART, DHAA, AA, and nACTs equivalent to the dosage of ART alone were dissolved in DMSO and sequentially diluted to different concentrations (5, 10, 20, 40, 80 μΜ) using DMEM as the medium. The final concentration of DMSO was 0.05% (v/v). Experiments were initiated by replacing the culture medium in each well with 100 μL of sample solutions at 37°C in the CO_2_ incubator. After 48 h of incubation, the medium was removed and 10 μL of CCK-8 reagent in the serum-free medium was added to each well. The plates were then incubated at 37°C for another 2 h. At the end of the incubation period, the plates were quantified by reading the absorbance at 450 nm on a micro-plate multi-detection instrument, SpectraMax iD3 with SoftMax® Pro (Molecular Devices Corporation Sunnyvale, CA, USA). The percentage of cell viability was calculated based on the absorbance measured relative to the absorbance of cells exposed to the negative control.

#### Transport studies.

10 μM of ART alone or its equivalent nACTs was added to well-cultured Caco-2, and the bidirectional permeability of ART from the apical (AP) to the basolateral (BL) side and from the basolateral to the AP side of the different forms of administration was measured directly by LC-MS/MS after 30, 60, 90, 120, 150, and 180 min.

The transport of R123 in normal Caco-2 cell monolayers or drug-treated Caco-2 cell monolayers was further studied. To prepare drug-treated Caco-2 cell monolayers, Caco-2 cells cultured in Transwell® inserts were aseptically treated on day 21 with 10 μM of ART, DEART, DHAA, AA and nACTs. Verapamil (Ver) is a known P-gp inhibitor and the reference uses a concentration of 100 μM as a positive control [36]. The transport buffer containing 5 μM of R123 was added on either the AP or the BL side of the inserts, as described above. At 30, 60, 90, 120, 150, and 180 min, an aliquot of 200 μL was withdrawn from the receiver chamber, respectively, and was immediately replenished with an equal volume of pre-warmed HBSS. The concentrations of R123 were immediately analyzed by SpectraMax iD3. The excitation wavelength was set at 485 nm, and the emission wavelength at 535 nm. P_app_ values across the cell monolayers were calculated according to the equation: P_app_ = dQ/dt × 1/(A × C_0_), where dQ/dt represents the rate of drug transport. A (1.12 cm^2^) is the effective surface area of the cell monolayer, and C_0_ (μM) is the initial drug concentration in the donor chamber [35]. The efflux ratio was calculated using the P_app_ (BL-AP) to P_app_ (AP-BL) ratio. P_app_ (AP-BL) represents the transport of ART from the apical to the basal side, and P_app_ (BL-AP) represents that from the basal to the apical side.

### Determination of artemisinin by LC-MS/MS

The LC-MS system consisted of an Agilent 1290 LC pump, an autosampler, and an Agilent 6490 triple quadrupole mass spectrometer (with electrospray ionization source). The separation of ART was performed on a Waters ACQUITY C18 Van Guard (2.1 mm × 5 mm, 1.7 μm) and a Waters ACQUITY UPLC BEH C18 column (2.1 mm × 100 mm, 1.7 μm). The mobile phase consisted of 0.1% formic acid aqueous solution (A) and acetonitrile (B). The solvent gradient was 65%B-95%B for 0–5 min; 95%B for 5–6 min; and 35%B for 6–9 min. The flow rate was 0.3 mL/min, and the column temperature was maintained at 30°C with an injection volume of 1 μL. The liquid chromatography system was coupled with an Agilent 6490 triple quadrupole mass spectrometer (USA) equipped with ESI ion source. The drying gas (N_2_) temperature was 200°C, the nitrogen flow rate was 15 L/min, the nebulizer pressure was 20 psi, the sheath gas temperature was 250°C, the sheath gas flow rate was 11 L/min, the capillary voltage was 3000 V, and the cleavage voltage was 380 V. The multi-reaction monitoring (MRM) channel was selected to be the m/z of artemisinin 283 → 247 m/z, and the collision energy was 10 eV.

### Statistical analysis

All data were presented as mean ± SD. Pharmacokinetic parameters including half-life (t_1/2_), maximum plasma time (T_max_) and concentration (C_max_), area under concentration-time curve (AUC_0-t_ and AUC_0-∞_), clearance (CL), steady-state volume of distribution (Vz), and mean residence time (MRT) were analyzed by non-compartmental method using DAS Version 2.0 (Chinese Pharmacological Society, Beijing, China). Statistical analyses were performed using SPSS Statistics software (version 16.0, IBM Analytics Inc., NY, USA) and GraphPad Prism software (version 8.0, GraphPad Software Inc., San Diego, CA, USA). The data were analyzed by Student’s t-test for two groups and one-way analysis of variance for multiple groups; *p* < 0.05 was considered statistically significant.

## Results

### The composition of *A. annua* extracts (nACTs)

Sesquiterpenes were isolated from *A. annua* extracts and their chemical structures were characterized by HRMS, NMR and comparison with previous studies. The molecular formula C_15_H_22_O_2_ was determined by HR-ESI-MS with m/z: 235.1800 [M + H]^+^ in positive ion mode (S3 FigA), and the ^1^H and ^13^C NMR data (S4 and S5 Figs) were consistent with the previous reports [37], identifying the compound as AA. Another HR-ESI-MS analysis in positive mode showed m/z: 237.1960 [M + H]^+^ (S3 FigB), indicating the formula C_15_H_24_O_2_, with ^1^H and ^13^C NMR data (S4 and S5 Figs) aligning with literature [38,39], identifying it as DHAA. HR-ESI-MS m/z: 235.1800 [M + H]^+^ (S3 FigC) reconfirms C_15_H_22_O_2_, and the ^1^H and ^13^C NMR data (S4 and S5 Figs) are in agreement with the literature [38,39], supporting its identification as ARTI. Finally, HR-ESI-MS provided m/z: 267.1716 [M + H]^+^ (S3 FigD), which led to the chemical formula of C_15_H_22_O_4_, and the ^1^H and ^13^C NMR data (S4 and S5 Figs) were in agreement with those reported in the literature [38,40], confirming that this is DEART. The identified sesquiterpene, after purification, showed a purity of over 98.4% using the HPLC normalization method.

**Fig 1:**
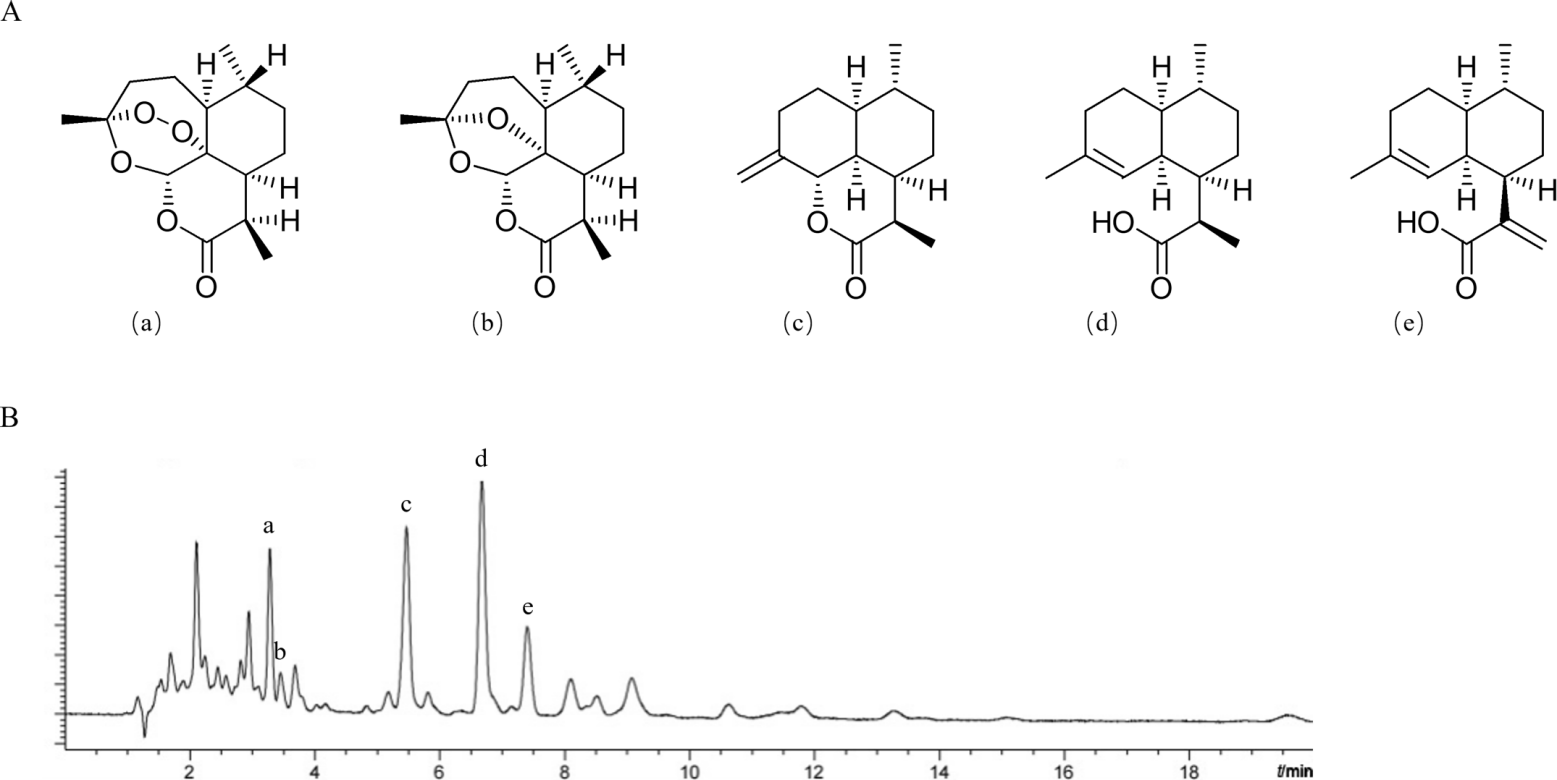
Chemical structures of five representative sesquiterpene compounds (A) and UPLC chromatograms (B). Note, (a) ART; (b) DEART; (c) ARTI; (d) DHAA; (e) AA.

In this study, *A. annua*, a high-quality resource with high ART content, was selected as the original medicinal material to obtain *A. annua* extracts (nACTs), a low-cost and dose-controlled natural antimalarial composition. During a routine extraction process with water, ART easily and quickly loses its efficacy, because the peroxide bridge structures are destroyed when the temperature exceeds 60°C. Therefore, we chose petroleum ether, a low-polarity solvent with a boiling range of 60–90°C in this study. After the petroleum ether refluxed extraction of *A. annua* plants, the extracts (nACTs) containing more than 50% sesquiterpenes were purified on silica gel columns. Five representative sesquiterpenes, namely ART, DEART, DHAA, AA and ARTI, were determined from nACTs by using the UPLC-DAD method (Fig 1 and Table 2). The contents of the five sesquiterpene components ART, DEART, ARTI, DHAA and AA in nACTs were 28.41%, 13.91%, 3.66%, 6.63% and 1.62% in that order. Among them, the content of ART reached more than 2% in the original herb of *A. annua*.

**Table 2:** Determination of five sesquiterpene components in nACTs and original herbs by UPLC-DAD method.

Compound	nACTs content (mg/g)	Original herbal content of *A.* annua (mg/g)
ART	284.1 ± 0.32	21.31 ± 0.41
DEART	139.1 ± 0.29	10.58 ± 0.11
DHAA	66.3 ± 0.11	5.18 ± 0.12
AA	16.2 ± 0.03	1.25 ± 0.02
ARTI	36.6 ± 0.02	2.95 ± 0.03

Note, each result is expressed as the mean ±SD, n=3.

### *In vivo* antimalarial efficacy

**Fig 2:**
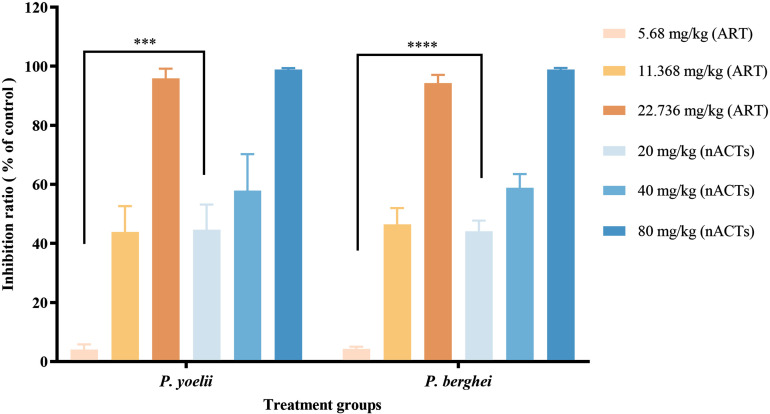
Anti-malarial efficacy of different treatment groups. Note, each column represents the mean ± SD (n = 5). Dose unit of administration is mg/kg. *** and **** indicate *p* < 0.001 and 0.0001, respectively.

*In vivo* antimalarial activity was assessed in mice infected with *P. yoelii* or *P. berghei* after oral administration of ART alone and nACTs. As shown in Fig 2, quantitative analysis showed that both medium-dose (40 mg/kg) and high-dose (80 mg/kg) nACTs treatment groups had better antimalarial efficacy than the corresponding ART groups at the same absolute ART dosage. However, no significant difference was observed. In contrast, there was a significant difference in the therapeutic efficacy at low doses. As can be seen from Fig 2, the antimalarial effect of low-dose ART (5.68 mg/kg) was poor, and could only achieve an inhibition rate of about 4%. However, at the same dosage of ART, the inhibition rate of nACTs (20 mg/kg) was about 42%, which was about 10-fold higher than that of ART. nACTs were a complex class of natural plant extracts containing more than 50% total sesquiterpenes. We hypothesized that the multiple components in nACTs might contribute to a higher antimalarial effect over ART alone at the same dosage of ART due to the synergistic effect.

### Bioanalytical method validation

In the specificity study, the retention times of ART, DEART, ARTI, DHAA, AA and buspirone were 4.64, 4.69, 5.39, 5.65, 5.78, and 1.50 min, respectively (S6 Fig). The endogenous substances and possible metabolites of blood did not interfere with the determination of each compound and the internal standard, and the substances to be measured and the internal standard did not interfere with each other’s determination. It was shown that the UHPLC-ESI-MS/MS method established in this study has good specificity. The linear ranges and standard curves for each compound are shown in Table 3, and the regression coefficients of the standard equations were all greater than 0.99, indicating that all standard curves met the requirements. The intra- and inter-batch precision RSDs of QC at low, medium, and high concentration levels were <15% and the accuracies were 85% ~ 115%, indicating that the method was qualified for precision and accuracy and meets the requirements for analytical testing of biological samples (S1 Table). Recoveries were in the range of 90.55% ~ 109.13%, indicating that the recoveries were consistent, precise, and reproducible. The matrix effect was in the range of 90.28% ~ 107.20%, which implied that no remarkable matrix effect was observed (S2 Table). Stability assessment showed that the RSD values (<15%) were good, implying no significant degradation of analytes under the tested conditions (S3 Table). The above results showed that the UHPLC-ESI-MS/MS method was fast and reliable, ensured the accuracy and reproducibility of the test results, and addressed the assessment of plasma drug concentrations at various time points post-administration.

**Table 3:** Linearity, range and lower limit of quantification of five compounds in rat plasma (n = 6).

Compound	Regression equation	Correlation coefficient (R)	Linear Range (ng/mL)	LLQ (ng/mL)
ART	*Y *= 5.053 × 10^-5^*X* - 8.922 × 10^–5^	0.9980	20 ~ 10000	20
DEART	*Y *= 4.371 × 10^-4^*X* - 7.270 × 10^–5^	0.9900	10 ~ 10000	10
ARTI	*Y *= 4.953 × 10^-4^*X *+ 3.232 × 10^–3^	0.9902	10 ~ 10000	10
DHAA	*Y *= 1.647 × 10^-5^*X *+ 8.841 × 10^–4^	0.9987	80 ~ 10000	80
AA	*Y *= 1.371 × 10^-5^*X *+* *9.498 × 10^–4^	0.9921	80 ~ 10000	80

### Pharmacokinetic study

**Fig 3:**
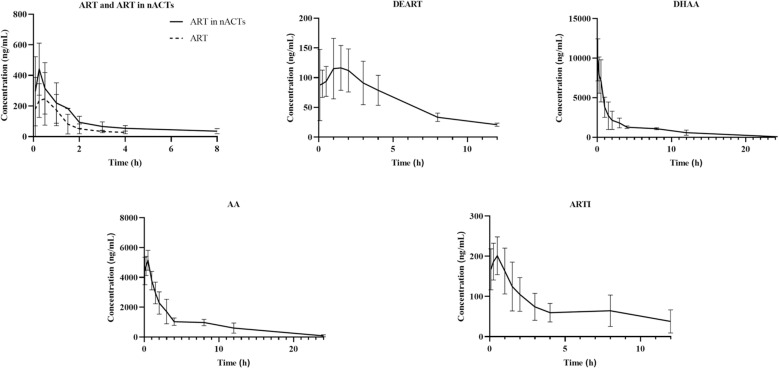
Pharmacokinetic profiles of representative ingredients.

The validated method for analysis of the five ingredients in rat plasma was then subjected to a pharmacokinetic study after oral administration of nACTs. The blood concentration-time curves of each compound after gavage administration of ART or nACTs are shown in Fig 3. The main pharmacokinetic parameters of each compound are shown in Table 4. The results showed that all five components could be detected in the blood, the plasma concentration-time curve was fitted, and pharmacokinetic parameters were calculated. Compared with rats given ART alone, the AUC_(0-t)_ of ART in nACTs group was increased by about 2-fold, the half-life was prolonged by about 1.6-fold, the mean retention time was prolonged, and the apparent volume of distribution and clearance was reduced. This indicates that the presence of other components in nACTs positively affects the bioavailability of ART in blood. In the nACTs administration group, the AUC_(0-t)_ and AUC_(0-∞)_ of AA and DHAA were higher, followed by ARTI and DEART. Notably, the bioavailability of DEART, ARTI, AA and DHAA was all higher than that of ART. The order of peak time of the five components was DEART > ARTI> ART > AA > DHAA, and the order of half-life was ARTI > DHAA > AA > DEART > ART.

**Table 4:** Pharmacokinetic parameters of the components of ART monomers and nACTs after gavage administration.

Parameters	ART (PU)	ART (nACTs)	DEART	ARTI	DHAA	AA
Dosage (mg/kg)	100.013	100.034	48.963	23.338	12.883	5.702
AUC_(0-t)_ (μg/L*h)	386.025 ± 128.460	763.464 ± 344.502	750.078 ± 113.819	989.72 ± 283.67	28711.335 ± 6659.186	22986.353 ± 4719.628
AUC_(0-∞)_ (μg/L*h)	418.286 ± 132.654	816.381 ± 382.936	848.072 ± 111.144	1386.162 ± 586.336	29716.411 ± 6699.016	23685.721 ± 4989.308
MRT_(0-t)_ (h)	1.175 ± 0.255	1.714 ± 0.513	4.045 ± 0.427	4.345 ± 0.957	5.392 ± 1.435	5.250 ± 1.237
MRT_(0-∞)_ (h)	1.673 ± 0.333	2.539 ± 0.785	6.123 ± 1.343	7.585 ± 3.947	6.077 ± 1.266	5.885 ± 1.614
t_1/2_ (h)	1.099 ± 0.335	1.759 ± 0.938	3.942 ± 1.569	5.145 ± 1.796	4.639 ± 1.183	4.289 ± 1.366
T_max_ (h)	0.625 ± 0.306	0.388 ± 0.328	1.375 ± 0.919	0.513 ± 0.51	0.22 ± 0.217	0.318 ± 0.208
CL (L/h)	0.052 ± 0.015	0.032 ± 0.018	0.024 ± 0.003	0.016 ± 0.006	0.001 ± 0.001	0.001 ± 0.001
Vz (L)	0.08 ± 0.03	0.061 ± 0.008	0.135 ± 0.049	0.114 ± 0.041	0.005 ± 0.001	0.005 ± 0.001
C_max_ (μg/L)	318.271 ± 147.512	513.229 ± 186.468	142.748 ± 37.277	221.674 ± 46.435	10386.656 ± 2444.647	5366.329 ± 589.8

*PU: Pure ART administration group; nACTs: *A. annua* extracts administration group. Note. The data are the average of three independent experiments and presented as the mean ± SD (n = 6).

### Cytotoxicity evaluation

**Fig 4:**
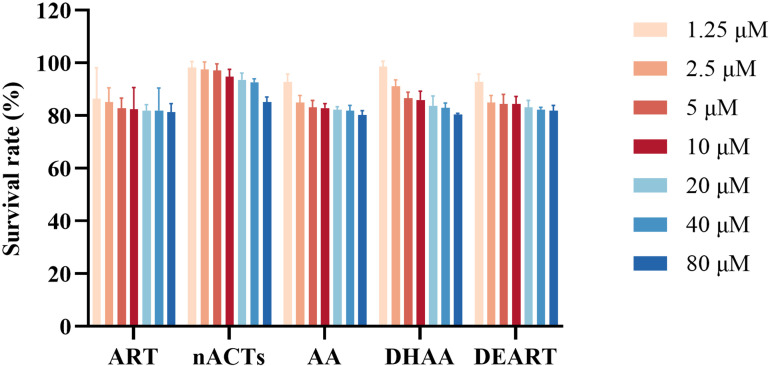
Determination of the effect of different concentrations of representative components on Caco-2 cell viability. Note, each column represents the mean ± SD (n = 6).

The cytotoxicity of ART, nACTs, DEART, AA, and DHAA on Caco-2 cells was determined by CCK-8 colorimetric assay. As shown in Fig 4, the cell survival rate showed a decreasing trend with the increase of the administered concentration in the range of 1.25–80 μM. However, the cell survival rates of all treatment groups cultured for 48 hours were greater than 80%, and no significant cytotoxicity was observed. From the perspective of environmental protection and safety, 10 μM was chosen as the final concentration for the following cell experiments.

### Caco-2 cell permeability assays to measure drug absorption

**Fig 5:**
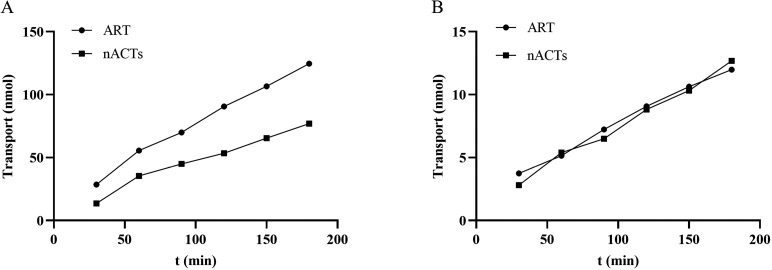
Time course of ART transport across Caco-2 cell monolayers. Note, Transepithelial transport of ART in the basolateral (BL)-to-apical (AP) direction (A) and in the apical (AP)-to-basolateral (BL) direction (B) across Caco-2 cell monolayers. 10 μM ART in ART alone or nACTs was added to the AP side or BL side. At predetermined time points, samples were withdrawn and the concentration was determined by LC-MS/MS. Each point represents the mean ± SD of (n = 3).

The transcellular transport of ART across a monolayer of Caco-2 cells over 3 hours is shown in Fig 5. The AP-to-BL and BL-to-AP permeabilities of ART were analyzed using LC-MS/MS for different routes of administration. The transcellular transport of ART from the AP side to the BL side (Fig 5B) and from the BL side to the AP side (Fig 5A) increased progressively over the 3 h period. When ART was in the form of a monomer or extract, its transport rate in the basolateral to apical (BL-AP) direction of the Caco-2 cell monolayer was much higher than that in the apical to basolateral (AP-BL) direction, and trans-epithelial transport was not saturated in either direction (Fig 5). As shown in Table 5, when ART was present in monomer form, the efflux ratio was 10.99, while that of ART in nACTs was 6.26. The efflux ratio of ART in extract form was significantly lower than that of ART alone, which implied that certain components of nACTs may affect the efflux of ART, thereby promoting increased intracellular drug concentrations.

**Table 5:** Characterization of ART transport in the Caco-2 cell model under different forms of drug administration.

Compound	P_app_ AP-BL (*10^–6^ cm/s)	P_app_ BL-AP (*10^–5^ cm/s)	Efflux Ratio (P_app_ BL-AP/P_app_ AP-BL)
ART (PU)	7.49 ± 0.35	8.23 ± 0.21*	10.99
ART (nACTs)	8.42 ± 0.37	5.27 ± 0.23*	6.26

P_app_ entries are mean ± SD (n = 3).

*Indicate *p *< 0.05 versus P_app_ AP-BL.

### Rhodamine 123 transport in Caco-2 cells

R123 is a classical substrate of P-gp and its transport can be inhibited by the P-gp-specific inhibitor Ver [19,41]. The efflux and translocation of R123 through the Caco-2 monolayer at different time points were measured to evaluate the effects of different drugs on P-gp function and expression. As shown in Table 6, Ver significantly inhibited the translocation of R123 from the BL side to the AP side from 13.55 to 3.13 (*p* < 0.01), compared with the untreated control. Meanwhile, the P-gp was inhibited by the activity, suggesting that the Caco-2 monolayer cell model was successfully established.

**Table 6:** Effects of drugs on the transport of R123 across Caco-2 cell monolayers.

Group	Concentration (μM)	P_app_ (10^–5 cm^/s)		
		BL-AP	AP-BL	Efflux Ratio
R123	5	4.27 ± 0.09	0.32 ± 0.85	13.55
R123 + ART	10	2.58 ± 0.39	0.25 ± 0.32	10.32*
	40	2.71 ± 0.42	0.24 ± 0.09	11.15*
	80	3.26 ± 0.23	0.26 ± 0.24	12.34
R123 + nACTs	10	2.32 ± 0.12	0.28 ± 0.16	8.25*
	40	2.40 ± 0.18	0.27 ± 0.36	8.86*
	80	2.46 ± 0.24	0.29 ± 0.12	8.45*
R123 + Ver	100	0.45 ± 0.08	0.14 ± 0.72	3.13**

The data are the average of six independent monolayers and presented as the mean ± SD (n = 3). * and ** Indicate *p* < 0.05 and 0.01 versus R123 group. Note, concentrations administered in the R123 + nACTs group indicate the addition of nACTs equivalent to 10, 40, and 80 μM of single-dose ART.

When different concentrations of ART monomers (80, 40 and 10 μM) were co-incubated with R123, the efflux ratios of R123 (12.34, 11.15 and 10.32) decreased significantly with decreasing concentrations of the administered drugs and showed a slight concentration dependence. However, whether the decrease in the Rho123 exocytosis ratio was due to ART acting as a substrate for P-gp, being substrate-competitive with Rho123, or affecting P-gp expression needs to be verified by further studies.

On the contrary, from the data in Table 6, it could be found that the nACTs (80, 40 and 10 μM) did not show concentration-dependence although they further reduced the efflux ratios of R123 (8.45, 8.86 and 8.25) compared with ART alone, implying that the involvement of multiple components in the nACTs could inhibit the efflux activity of P-gp. The preliminary results suggest that the potentiating effect of nACTs may be related to drug transport and absorption. As a complex herbal extract, the interaction between substrate, inhibitor, and transporter proteins would be more complicated.

**Fig 6:**
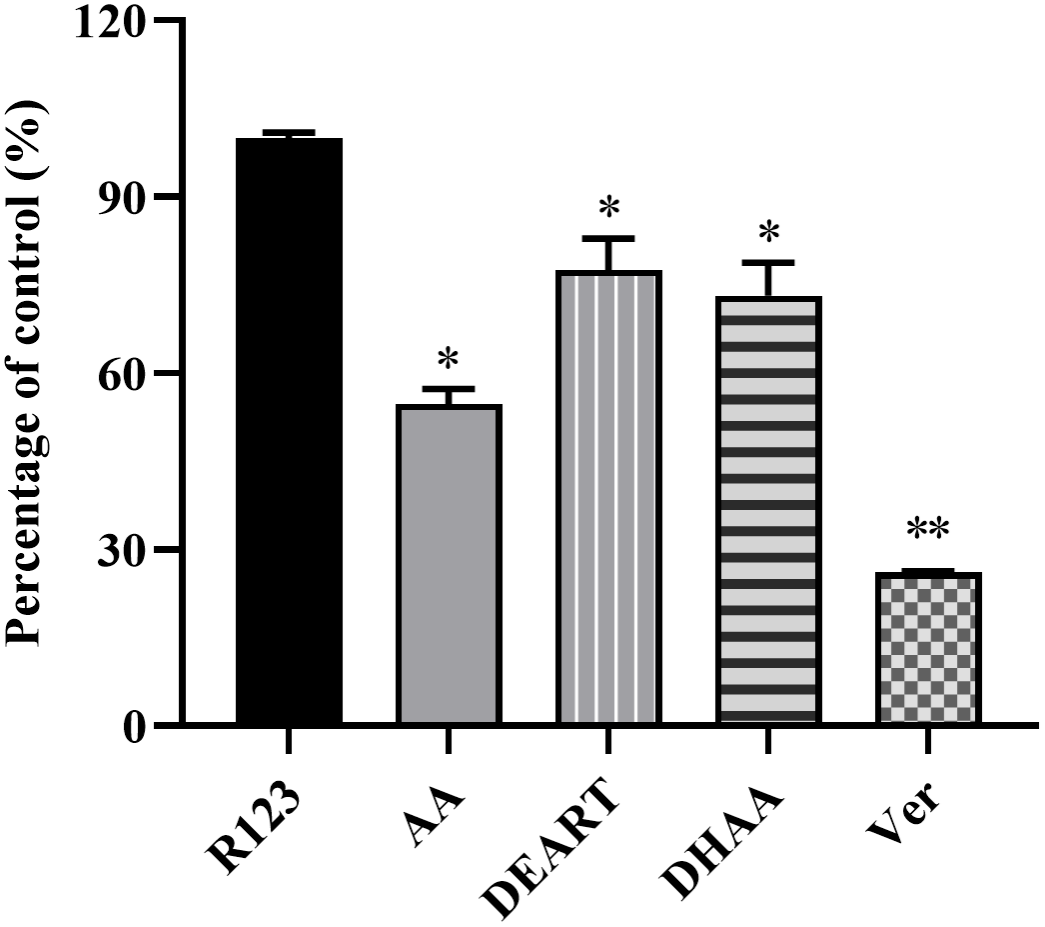
Screening for the inhibition of P-glycoprotein mediated Rhodamine-123 transport by AA, DEART, DHAA and Ver. Note, each group n = 3; * and ** indicate *p* < 0.005 and 0.001, respectively.

To further investigate in-depth, the effects of different components on the P-gp function, the representative components DEART, DHAA, and AA were measured. As shown in Fig 6, when DEART, DHAA and AA were co-incubated with R123, they were each found to inhibit the efflux and translocation of P-gp on the substrate R123, which was similar to Ver. When DEART, DHAA or AA was combined with R123, the aggregation of R123 from the BL side to the AP side was reduced by 22.40%, 45.21% and 26.82%, respectively. The results suggest that, in addition to ART, the presence of other components such as DEART, DHAA and AA in *A. annua* extracts may have an inhibitory effect on the efflux effect of P-gp.

## Discussion

It is well known that ART, a sesquiterpenoid, has some antimalarial effects. In recent years, more and more researchers have focused on the antimalarial activity of *A. annua* multicomponents in malaria treatment, but the considerable production cost and insufficient ART content have limited its current role in the campaign against malaria. There is now much evidence that other components of *A. annua* may have a direct or indirect effect on ART bioavailability. Previous studies have found that administration of ART in the form of dried *A. annua* leaves and *A. annua* tea (*A. annua* hot water infusion) significantly improves the bioavailability and efficacy of ART [12,42–44]. In addition, Elfawal and Weathers’ team found that *A. annua* had more significant antimalarial efficacy than ART alone at the same dose. Compared to ART alone, mice receiving dried *A. annua* leaves orally showed a 5-fold increase in blood clearance of malaria, more bioavailable in blood than ART monomer compounds, and even a 3-fold increase in slowing the development of malaria resistance [10,45,46]. The group found that the combination of arteannuin B, AA, scopoletin, and ART can improve the efficacy and bioavailability of ART, which provides a possibility to develop new natural multi-component artemisinin-based combination therapies [17]. However, obtaining or preparing these compounds in resource-limited malaria-endemic areas is very challenging. Therefore, the four distribution ratios are not easily achievable in malarial areas. In this study, *A. annua*, a resource with high ART content that has been cultivated and bred on a large scale in China for more than four decades, was utilized in the context of multicomponent synergistic effects, and extracted and purified using a low-polarity solvent, petroleum ether. Thus, a plant-based natural antimalarial drug, *A. annua* extract, was obtained, which is characterized by low cost, controllable dosage and remarkable efficacy.

Soybean oil was used as a solvent to fully dissolve the drug and avoid the problem of poor solubility of ART. The antimalarial activity of nACTs was evaluated using two murine malaria models, *P. yoelii* and *P. berghei*, and the results showed that the antimalarial efficacy of nACTs was significantly better than that of ART alone at the same dosage, with an improvement of nearly 10-fold (Fig 2). This implies that synergistic antimalarial effects may have been produced by the interaction of multiple components in the herb, providing a theoretical basis for the further development of new natural multicomponent antimalarial drugs.

The pharmacokinetic characteristics of *A. annua* extract after oral administration were further investigated by *in vivo* experiments. Significant increases in AUC and C_max_ were observed for ART when administered as nACTs compared to ART alone, demonstrating the beneficial effects of the combination. It has also been found that delivery of ART in the form of tea has a shorter time to peak compared to ART alone, reflecting a more favorable drug absorption in the form of tea preparations [47]. nACTs are a class of *A. annua* extracts with a complex composition, and the interactions between different components add more to the many mechanisms of bioavailability enhancement. Previous studies have demonstrated that AA, arteannuin B, and scopoletin may affect the binding of ART to serum albumin in the blood circulation, thereby altering the distribution, metabolism, and excretion of ART [17,48]. In addition, ART undergoes extensive metabolism, which is mediated by cytochrome P450 metabolizing enzymes, particularly CYP2B6 and CYP3A4 [49,50]. It has been shown that arteannuin B is an inhibitor of CYP3A4 and that the AUC and therapeutic effect of ART can be enhanced by metabolism-dependent synergism with arteannuin B [51]. In the present study, the nACTs group had a higher AUC, a longer t_1/2_, and a lower CL, indicating inhibition of metabolism and excretion, which is also consistent with previous reports [51,52]. The prolonged exposure and duration of action suggest that the potentiating effect of nACTs may be related to the improved pharmacokinetic behavior of ART in them. The peak concentrations and bioavailability of AA and DHAA were much higher than those of the three regional components in the study, and it is inferred that the carboxyl side chains in the structure of both compounds probably play an important role. It is noteworthy that the gavage administration of ART is generally carried out using a suspension of ART prepared with CMC-Na due to its physicochemical properties, whereas in our study, an oil solution was given in which ART is in a dissolved state. This may lead to faster absorption of ART and its derivatives, which is also reflected in the results of the study compared to those of the peers [12], and the peak time of ART was detected relatively earlier in this study.

In the *in vitro* experiments, the effect of nACTs on P-gp substrate uptake and translocation in Caco-2 cells was first investigated. Since ART is a sesquiterpenoid containing a peroxy-bridge structure [53,54], the lack of a conjugation system in the molecular structure makes it difficult to quantify biological samples with conventional UV detectors. In this study, R123 was chosen as a fluorescent marker to indirectly explain the effect of ART and other components on P-gp.

In this experiment, the changes of ART content on both sides of Caco-2 cells under different administration forms of ART monomer and extract were directly detected by LC-MS/MS, and it was found that the proportion of ART efflux was significantly higher than that of the extract when administered in the form of monomer (Fig 5 and Table 5). This may be due to the presence of components in the extract that affect ART efflux. The combined effect of multiple components in nACTs reduced ART efflux and thus increased the intracellular drug concentration, which might also be one of the reasons for the better antimalarial efficacy of nACTs. Further, R123 was used to detect whether Caco-2 cell monolayers expressed P-gp and the effects of ART and nACTs on P-gp activity were investigated in depth. It was found that both ART and nACTs reduced the efflux ratio of R123 (Table 6), which indicated that they exerted some inhibitory effects on P-gp within the concentration range of the experimental study, consistent with the results of previous studies [55].

Further, co-incubation of the representative components DEART, DHAA and AA with Caco-2 cells revealed that they all possessed properties similar to Ver, inhibiting the efflux and translocation of P-gp to the substrate R123. These components are present in the extract along with ART, and the interaction of the different components reduces the efflux of ART, which may also account for the increased bioavailability of ART in the extracts. The interaction of multiple components in *A. annua* extracts increased ART bioavailability and produced better antimalarial activity *in vivo*. The reported improved sensitivity of drug-resistant *P. berghei* k173 to ART with artemisinin-chrysosplenetin combination may be because ART activates the expression of intestinal P-gp and Abcb1/Abcg2 and inhibits the expression of breast cancer resistance protein, whereas chrysosplenetin reverses these expressions [56]. Co-administration of antimalarial P-gp substrate drugs with antimalarials that act as P-gp inhibitors increases the oral bioavailability of the former, thereby reducing the dose required to clear the infection [57]. This also offers the possibility of reducing treatment costs as well as preventing the emergence and spread of drug resistance. Also understanding the affinity of antimalarials for efflux transporter proteins such as P-gp may help characterize drug absorption and pharmacokinetic drug interactions. For example, it was found that mefloquine is a P-gp inhibitor and methylene blue is a partial substrate; if co-administered with such P-gp inhibitors, methylene blue absorption is increased [58]. Such as flavonoids that have been found to act as P-gp inhibitors, which can affect the oral bioavailability of the drug by altering the level of P-gp [59,60]. Moreover, flavonoids can also affect P-gp configuration by directly interacting with adjacent ATP-binding sites and steroid-binding sites, substrate-binding domains, or anisotropic mechanisms [61]. It was also found that chrysosplenetin inhibited P-gp-mediated ART efflux by reversing ART-induced up-regulation of MDR1 mRNA and P-gp expression [62].

nACTs are a class of *A. annua* extracts containing a variety of complex compounds such as sesquiterpenes and flavonoids, the components of which form an organic whole acting together to produce more significant advantages than the antimalarial effects of ART, which also reflects the complexity and holistic nature of Chinese medicine. Other components of *A. annua* can have direct or indirect effects on ART bioavailability by affecting metabolism. It has been found that arteannuin B inhibits CYP3A4 with an IC_50_ of 1.2 μM and enhances the antimalarial efficacy of ART by regulating the metabolism of ART [51]. Noncompetitive or uncompetitive inhibition of drug-metabolizing enzymes by the flavonoid chrysosplenetin contributes to the synergistic antimalarial effect of ART [63]. Dried leaves of *A. annua* were found to enhance the bioavailability and downstream efficacy of ART by inhibiting cytochrome P450 [64]. The digestive material of *A. annua* dried leaves can also increase the intestinal permeability of ART [65].

*Artemisia afra*, an *Artemisia* spp. plant with very low ART content has a long history of medicinal use in southern Africa, but a recent study found that an aqueous powdered suspension of *A. annua* had antimalarial activity in mice, but *Artemisia afra* did not [66]. This is more in part a reflection of the fact that complex plant extracts containing a certain amount of ART are a more cost-effective way to stop this deadly disease [67]. Compared to the previous approach using a four-component combination therapy of ART, artemisinin B, artemisinic acid, and scopoletin (1:1:1:1), which posed challenges due to high costs and logistical difficulties in malaria-endemic regions, nACTs offer a more practical and cost-effective alternative [17]. We believe that *A. annua* multi-component has the advantage of synergistic effect and is more worthy of attention. Petroleum ether extraction and silica gel column purification were used to obtain nACTs enriched with sesquiterpenoids, which provided good control of ART dosage, an easy extraction method, favorable price, and higher bioavailability. Due to environmental conditions, cultivation methods, harvesting seasons, and genetic diversity different geographic sources of *A. annua* have different types of chemical constituents and varying concentrations of bioactive compounds, thus affecting the reproducibility and consistency of *in vivo* studies [68–70]. However, after years of breeding and propagation, the ART content of *A. annua* grown in the Hunan region of China is above 2%. We found that the quality of *A. annua* was stable after collecting several batches of *A. annua* for measurement in the early stage. *A. annua* grown in a specific region was enriched with sesquiterpenoids by industrialized extraction using silica gel decontamination, which greatly ensured the consistency and stability of nACTs. The content of ART in nACTs can be controlled, which greatly ensures the stability of ART dosage. In addition, the antimalarial effect of nACTs is more significant than that of ART alone, with better bioavailability. In the future, nACTs can be developed into soft gelatin capsules for industrial production, which is convenient for dosage control and clinical use.

This study *in vitro* Caco-2 cell studies revealed that some components of the extract (DEART, AA and DHAA) may inhibit ART efflux by inhibiting P-gp efflux and translocation. Quantitative analysis revealed that nACTs contained more than 50% of the five sesquiterpenoids, ART, DEART, DHAA, AA and ARTI. Future experiments should explore the presence of non-artemisinin-based antimalarial active ingredients in the remaining unknown constituents other than the five, as well as their mechanisms of interaction with ART *in vivo*.

## Conclusion

The antimalarial efficacy of nACTs was found to be significantly increased by approximately 10-fold compared to ART alone at the same ART dosage in two malaria mouse models (*P. yoelii* and *P. berghei*). In addition, administration of the extract increased the bioavailability, half-life, mean retention time and peak concentration of ART, as well as decreased the apparent volume of distribution and clearance. This provided strong evidence of potential synergistic antimalarial effects among multiple components of *A. annua* herbal extract. In addition, further *in vitro* Caco-2 cell experiments showed that the components of the extract (DEART, AA and DHAA) inhibited the efflux and translocation of P-gp and inhibited the efflux of ART, which may explain the synergistic antimalarial effect of ART with these components.

## Supporting information

S1 Fig Mass spectra of each target compound. (TIF)

S2 Fig Possible cleavage patterns of qualitative ions of target compounds over quantitative ions. Note, 1. ART qualitative ion; 2. ART quantitative ion; 3. DEART qualitative ion; 4. DEART quantitative ion; 5. ARTI qualitative ion; 6. ARTI quantitative ion; 7. DHAA qualitative ion; 8. DHAA quantitative ion; 9. AA qualitative ion; 10. AA quantitative ion.
(TIF)

S3 Fig High-resolution mass spectra of four sesquiterpenoids. The [M + H]^+^ ion peaks of AA (A), DHAA (B), ARTI (C) and DEART (D) were identified in the positive ion mode.
(TIF)

S4 Fig ^1^H-NMR of four sesquiterpenoids. Note, (A) AA; (B) DHAA; (C) ARTI; (D) DEART.
(TIF)

S5 Fig ^1^C-NMR of four sesquiterpenoids. Note, (A) AA; (B) DHAA; (C) ARTI; (D) DEART.
(TIF)

S6 Fig UHPLC-ESI-MS/MS method for the determination of five compounds in SD rat plasma exclusively. Note, (A) blank plasma; (B) plasma samples from rats after drug administration; (C) plasma at the LLOQ concentration level; and (D) plasma at the MQC concentration. Note. B, C, D graphs from top to bottom the arrows point to ART, DEART, ARTI, DHAA and buspirone in that order.
(TIF)

S1 Table Precision and accuracy results of five compounds in rat plasma (n = 5). (DOCX)

S2 Table Matrix effect and recovery measurements of five compounds in rat plasma (n = 5). (DOCX)

S3 Table Results of stability studies of five compounds in SD rat plasma using UHPLC-ESI-MS/MS (n = 5). (DOCX)

S1 File ^1^H and ^13^C NMR data for four sesquiterpenoids. (PDF)

S2 File Data related to the experiment. (ZIP)

## Abbreviations

AA: Artemisinic acid
ACTs: Artemisinin-based combination therapies
AP: Apical side
ART: Artemisinin
ARTI: Arteannuin I
AUC: area under the concentration-time curve
BL: Basolateral side
C_max_: peak concentration
Caco-2 cells: human colorectal adenocarcinoma cell
CCK-8: Cell counting kit-8
CL_Z_: clearance rate
DEART: Deoxyartemisinin
DHAA: Dihydroartemisinic acid
DMSO: Dimethyl sulfoxide
DMEM: Dulbecco’s modified eagle’s medium
ED_50_: median effective dose
ER: efflux ratio
HBSS: Hank’s balanced salt solution
LC-MS: Liquid Chromatography-Mass Spectrometry
LLOQ: lower limit of quantification
MRT: mean resident time
nACTs: natural ACTs/*A. annua* extracts
P_app_: Apparent permeability coefficient
P-gp: P-glycoprotein
PBS: Phosphate buffer solution
RSD: relative standard deviation
R123: rhodamine123
T_max_: peak time
t_1/2_: Elimination half-life
UPLC: Ultra Performance Liquid Chromatography
V_Z_: apparent volume of distribution
Ver: Verapamils
