# Supplementary figures and images for: The plant matrix of Artemisia annua L. for the treatment of malaria: Pharmacodynamic and pharmacokinetic studies
Source: PLoS One. 2025 May 7;20(5):e0322835. doi: 10.1371/journal.pone.0322835 (PMC12058161; doi:10.1371/journal.pone.0322835)

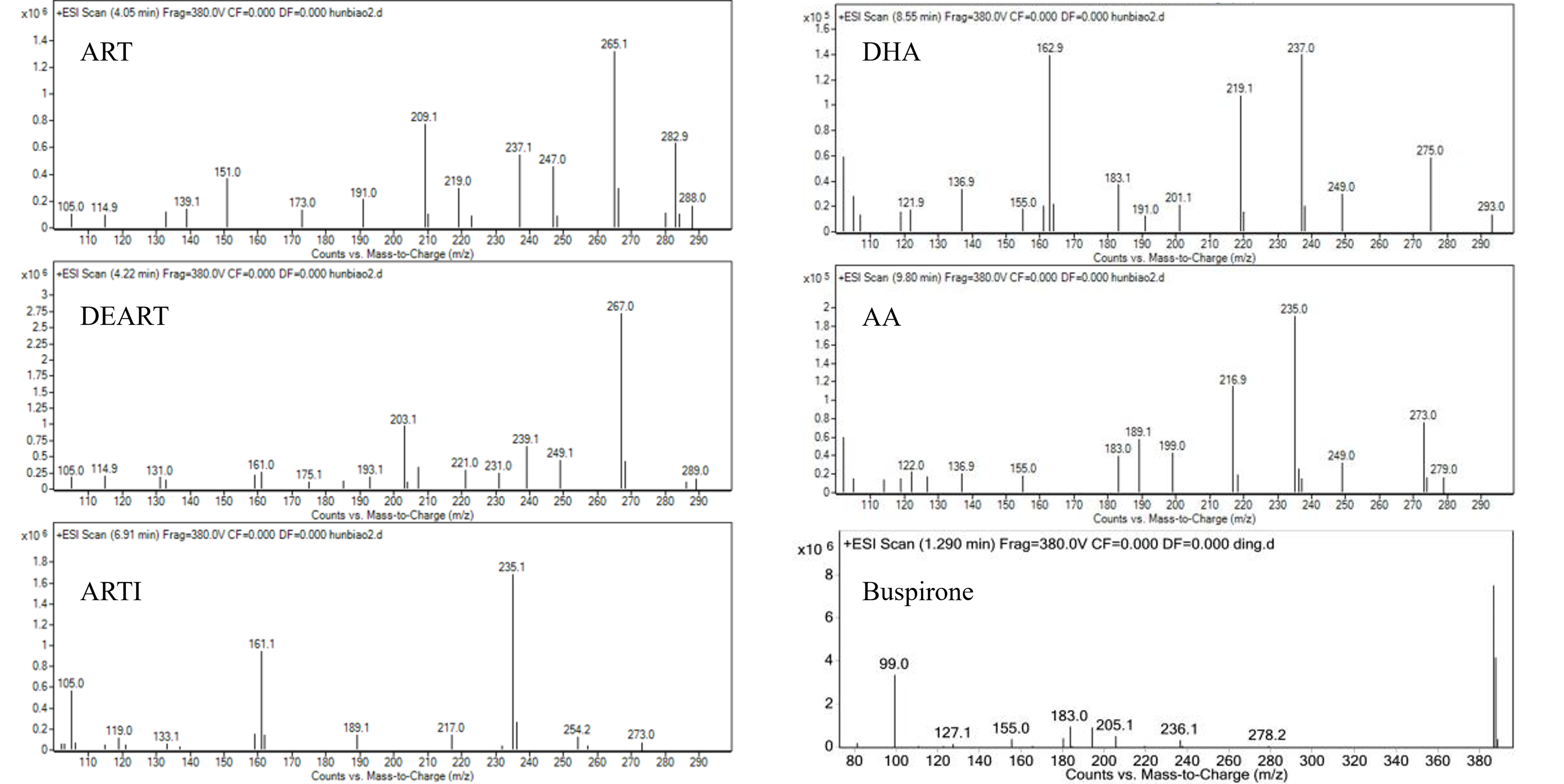

Supplement: S1 Fig — (TIF) [file pone.0322835.s001.tif]

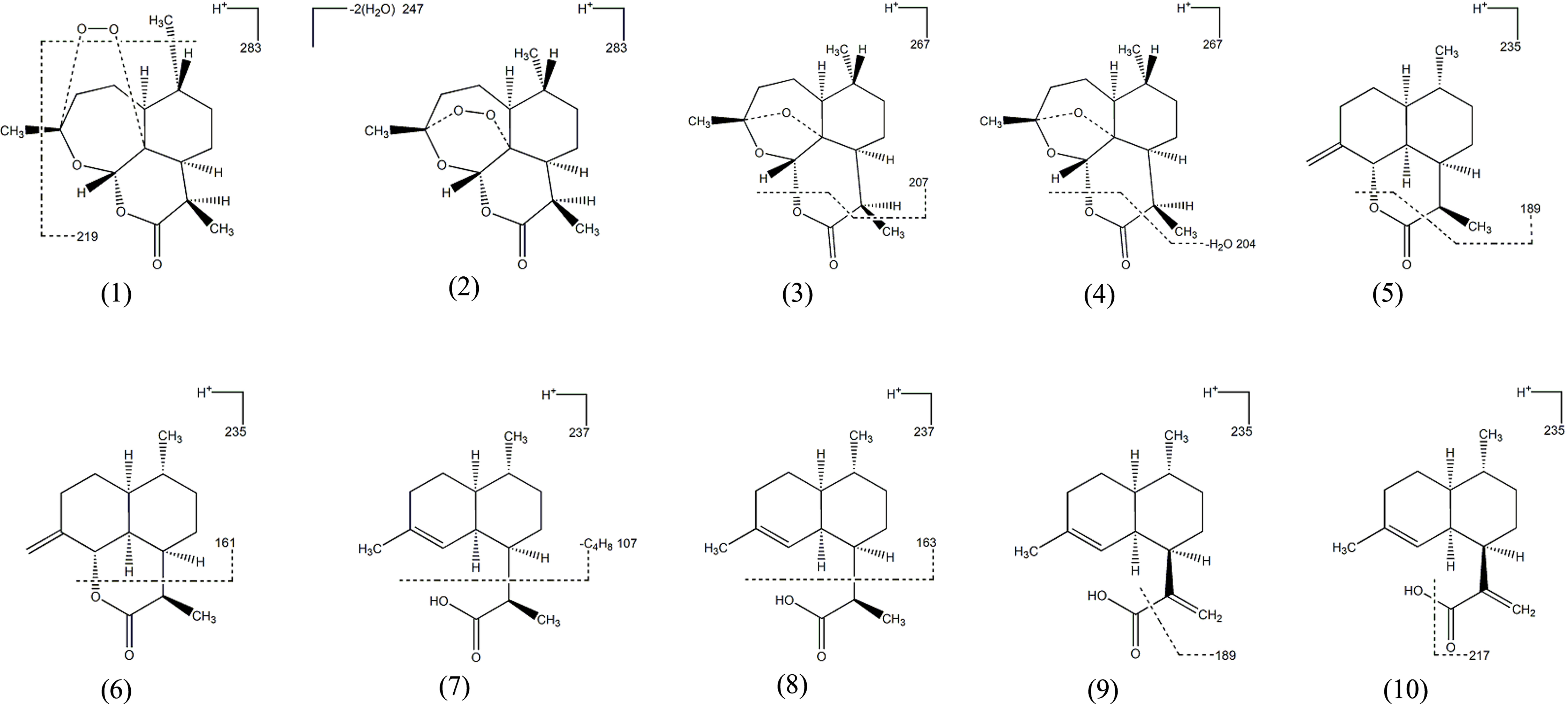

Supplement: S2 Fig — Note, 1. ART qualitative ion; 2. ART quantitative ion; 3. DEART qualitative ion; 4. DEART quantitative ion; 5. ARTI qualitative ion; 6. ARTI quantitative ion; 7. DHAA qualitative ion; 8. DHAA quantitative ion; 9. AA qualitative ion; 10. AA quantitative ion. (TIF) [file pone.0322835.s002.tif]

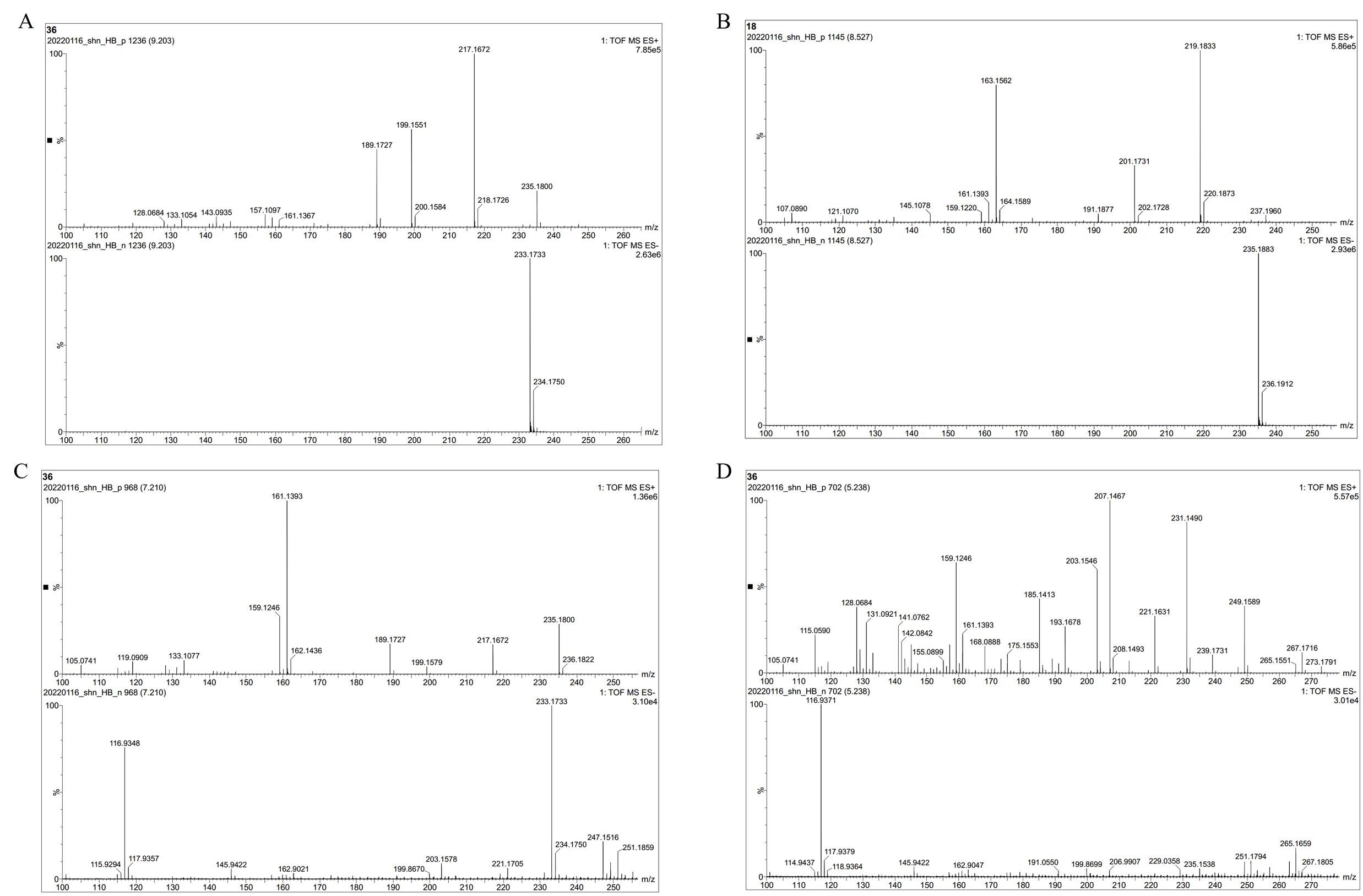

Supplement: S3 Fig — The [M + H]+ ion peaks of AA (A), DHAA (B), ARTI (C) and DEART (D) were identified in the positive ion mode. (TIF) [file pone.0322835.s003.tif]

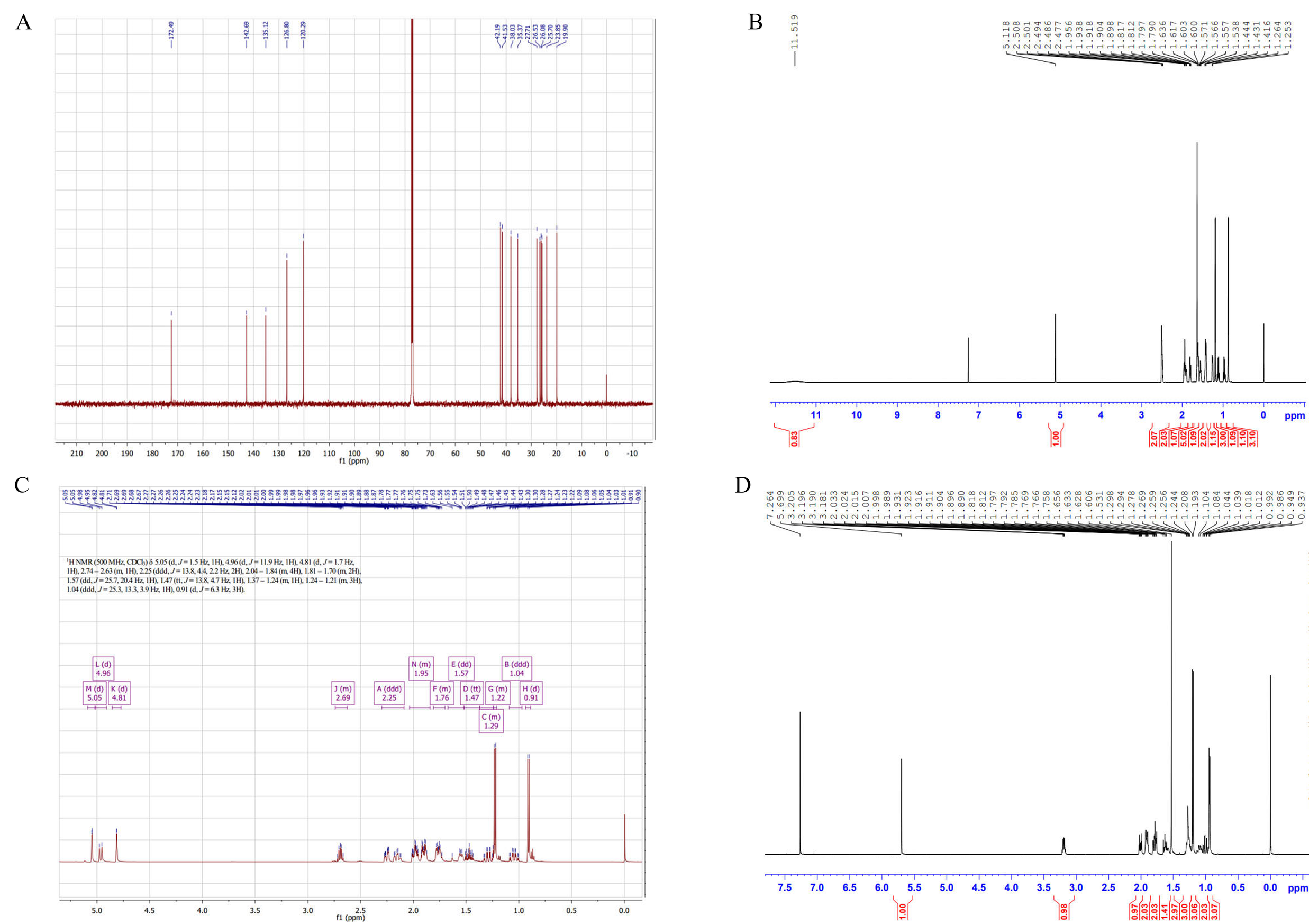

Supplement: S4 Fig — Note, (A) AA; (B) DHAA; (C) ARTI; (D) DEART. (TIF) [file pone.0322835.s004.tif]

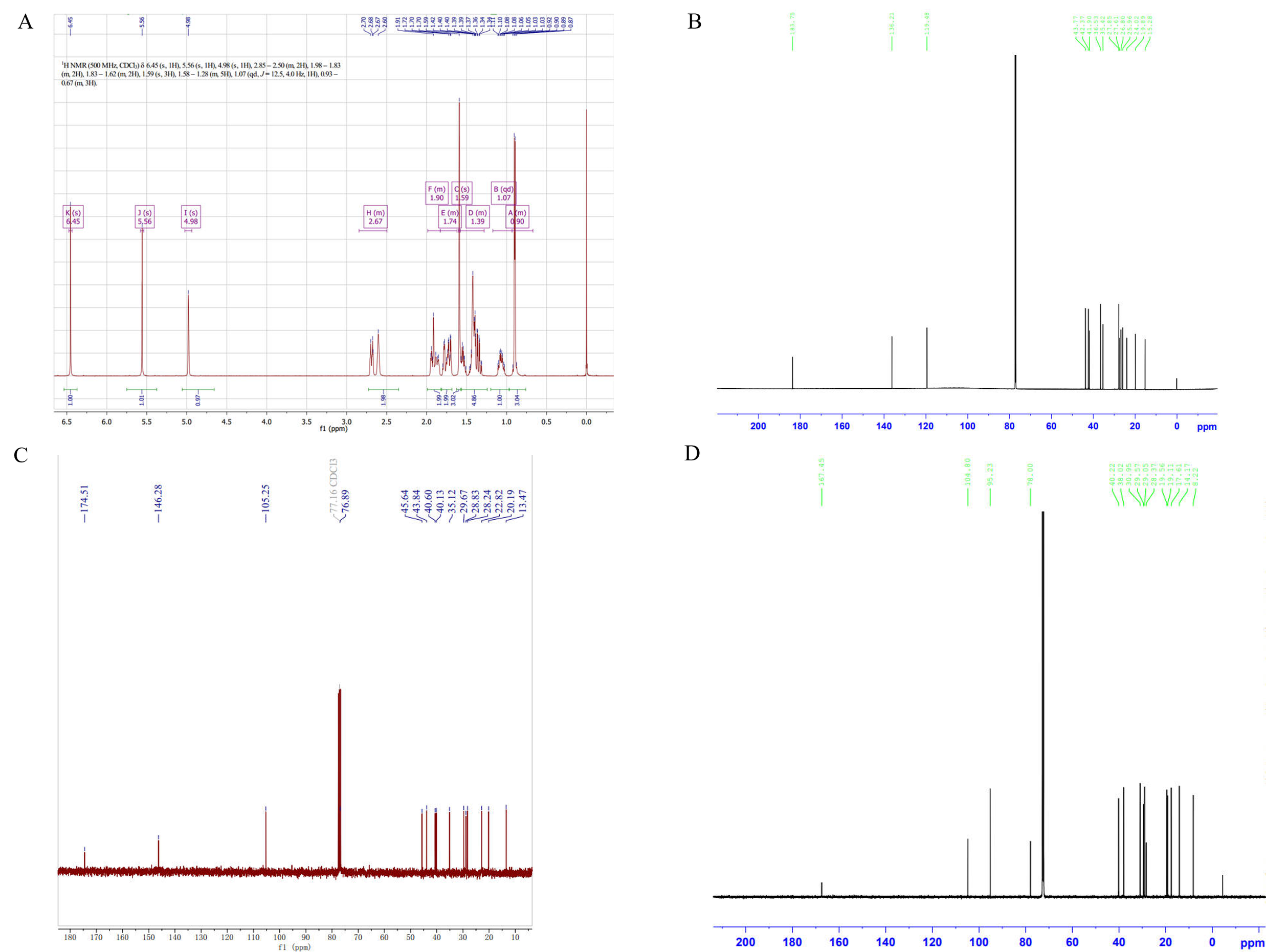

Supplement: S5 Fig — Note, (A) AA; (B) DHAA; (C) ARTI; (D) DEART. (TIF) [file pone.0322835.s005.tif]

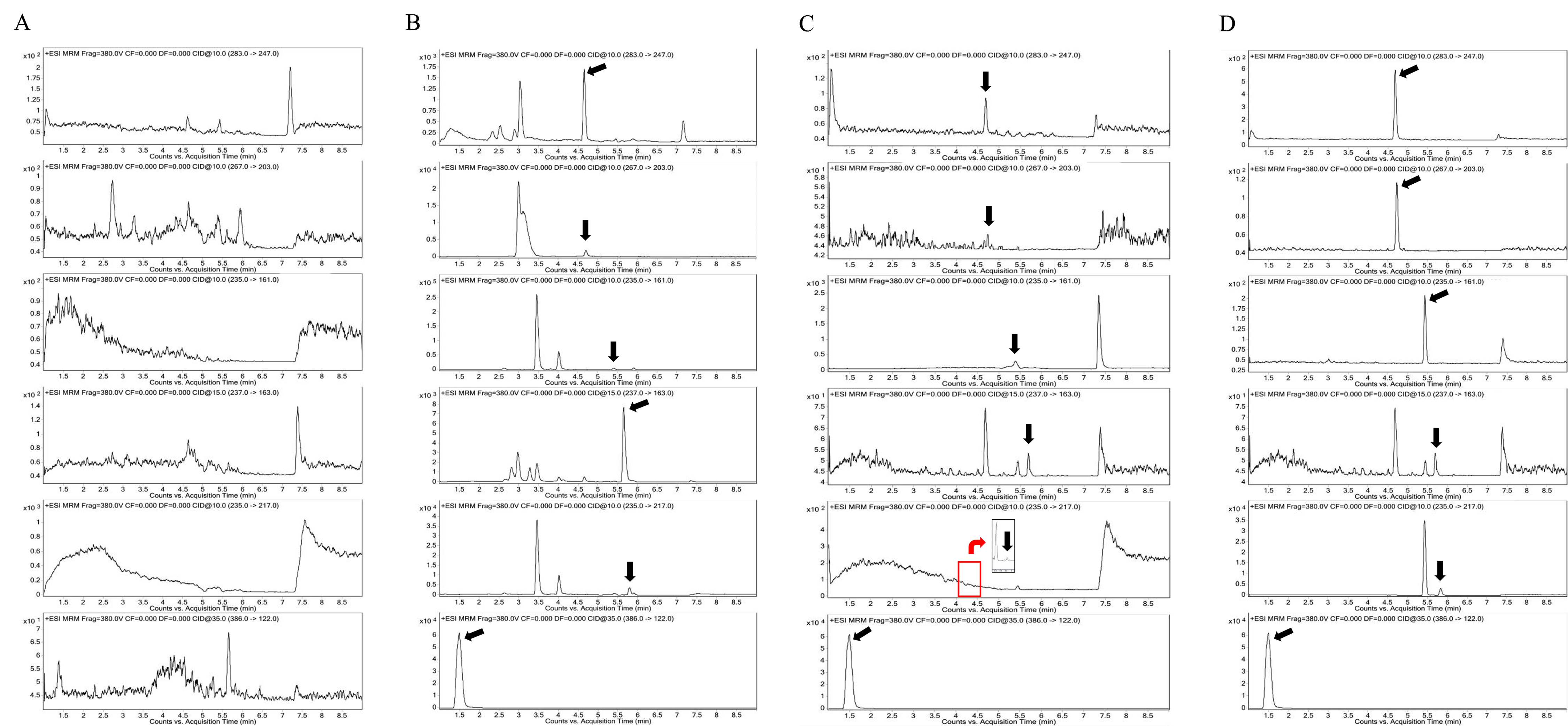

Supplement: S6 Fig — Note, (A) blank plasma; (B) plasma samples from rats after drug administration; (C) plasma at the LLOQ concentration level; and (D) plasma at the MQC concentration. Note. B, C, D graphs from top to bottom the arrows point to ART, DEART, ARTI, DHAA and buspirone in that order. (TIF) [file pone.0322835.s006.tif]
