# Supplementary material for: The plant matrix of Artemisia annua L. for the treatment of malaria: Pharmacodynamic and pharmacokinetic studies
Source: PLoS One. 2025 May 7;20(5):e0322835. doi: 10.1371/journal.pone.0322835 (PMC12058161; doi:10.1371/journal.pone.0322835)
Supplement: S1 Table — (DOCX) [file pone.0322835.s007.docx]

**S1 Table Precision and accuracy results of five compounds in rat plasma (n=5)**

| Compound | Concentration  (ng/mL) | Batch 1 | | | Batch 2 | | | Batch 3 | | | Inter-batch precision | | |
| --- | --- | --- | --- | --- | --- | --- | --- | --- | --- | --- | --- | --- | --- |
|  |  | mean ± SD | RSD  (%) | Accuracy | mean ± SD | RSD  (%) | Accuracy | mean ± SD | RSD  (%) | Accuracy | mean ± SD | RSD  (%) | Accuracy |
| ART | 40 | 38.9 ± 2.40 | 6.17% | 97.23% | 41.7 ± 4.04 | 9.70% | 104.27% | 38.6 ± 1.49 | 3.87% | 96.43% | 39.7 ± 1.72 | 4.34% | 99.31% |
|  | 1000 | 1060 ± 14.3 | 1.34% | 105.99% | 979 ± 66.4 | 6.79% | 97.90% | 1061 ± 31.1 | 2.93% | 106.10% | 1033 ± 47.04 | 4.55% | 103.33% |
|  | 5000 | 4666 ± 174 | 3.73% | 93.32% | 5383 ± 461 | 8.57% | 107.65% | 4707 ± 191 | 4.05% | 94.13% | 4918 ± 403 | 8.19% | 98.37% |
| DEART | 20 | 19.3 ± 0.84 | 4.36% | 96.30% | 20.8 ± 1.46 | 7.05% | 103.81% | 20.6 ± 1.68 | 8.19% | 102.76% | 20.2 ± 0.81 | 4.03% | 100.96% |
|  | 1000 | 1061 ± 30 | 2.84% | 106.12% | 1065 ± 93.6 | 8.78% | 106.52% | 1016 ± 41.9 | 4.13% | 101.55% | 1047 ± 27.6 | 2.64% | 104.73% |
|  | 5000 | 4987 ± 145 | 2.91% | 99.74% | 5079 ± 300 | 5.91% | 101.57% | 4593 ± 188 | 4.09% | 91.86% | 4886 ± 258 | 5.28% | 97.72% |
| ARTI | 20 | 19.6 ± 1.75 | 8.93% | 97.95% | 21.4 ± 1.66 | 7.74% | 107.06% | 20.5 ± 1.30 | 6.33% | 102.37% | 20.5 ± 0.91 | 4.45% | 102.46% |
|  | 1000 | 1010 ± 39.8 | 3.94% | 101.02% | 1076 ± 97 | 9.02% | 107.63% | 1054 ± 14.5 | 1.38% | 105.43% | 1047 ± 33.6 | 3.21% | 104.70% |
|  | 5000 | 4913 ± 174 | 3.53% | 98.27% | 5349 ± 379 | 7.09% | 106.98% | 4759 ± 295 | 6.20% | 95.18% | 5007 ± 306 | 6.11% | 100.14% |

**Continued S1 Table Precision and accuracy results of five compounds in rat plasma (n=5)**

| Compound | Concentration  (ng/mL) | Batch 1 | | | Batch 2 | | | Batch 3 | | | Inter-batch precision | | |
| --- | --- | --- | --- | --- | --- | --- | --- | --- | --- | --- | --- | --- | --- |
|  |  | mean ± SD | RSD  (%) | Accuracy | mean ± SD | RSD  (%) | Accuracy | mean ± SD | RSD  (%) | Accuracy | mean ± SD | RSD  (%) | Accuracy |
| DHAA | 160 | 155 ± 6.90 | 4.46% | 96.57% | 172 ± 11.3 | 6.57% | 107.60% | 166 ± 8.08 | 4.88% | 103.52% | 164 ± 8.92 | 13.03% | 102.56% |
|  | 1000 | 1062 ± 60.5 | 5.70% | 106.29% | 1056 ± 66.6 | 6.30% | 105.60% | 1096 ± 19.7 | 1.80% | 109.60% | 1072 ± 21.4 | 14.09% | 107.17% |
|  | 5000 | 5184 ± 211 | 4.06% | 103.68% | 5008 ± 377 | 7.53% | 100.15% | 4884 ± 224 | 4.59% | 97.67% | 5025 ± 151 | 3.01% | 100.50% |
| AA | 160 | 154 ± 16.0 | 10.40% | 95.96% | 170 ± 14.0 | 8.24% | 106.12% | 166 ± 13.5 | 8.15% | 103.76% | 163.1 ± 8.51 | 5.21% | 101.95% |
|  | 1000 | 1036 ± 44.0 | 4.25% | 103.60% | 980 ± 48.2 | 4.91% | 98.05% | 1028 ± 19.9 | 1.94% | 102.78% | 1015 ± 30.0 | 2.95% | 101.47% |
|  | 5000 | 4985 ± 224 | 4.48% | 99.69% | 5026 ± 383 | 7.61% | 100.52% | 4799 ± 190 | 3.96% | 95.99% | 4937 ± 121 | 2.45% | 98.73% |
