# Supplementary material for: The plant matrix of Artemisia annua L. for the treatment of malaria: Pharmacodynamic and pharmacokinetic studies
Source: PLoS One. 2025 May 7;20(5):e0322835. doi: 10.1371/journal.pone.0322835 (PMC12058161; doi:10.1371/journal.pone.0322835)
Supplement: S2 Table — (DOCX) [file pone.0322835.s008.docx]

**S2 Table Matrix effect and recovery measurements of five compounds in rat plasma (n=5)**

| Compound | Concentration (ng/mL) | matrix effect (%) | RSD (%) | Recovery (%) | RSD (%) |
| --- | --- | --- | --- | --- | --- |
| ART | 40 | 107.20 | 5.31 | 109.13 | 5.70 |
|  | 1000 | 102.55 | 8.59 | 99.07 | 3.36 |
|  | 5000 | 107.10 | 7.50 | 106.04 | 1.98 |
| DEART | 20 | 101.41 | 5.41 | 108.62 | 6.62 |
|  | 1000 | 104.97 | 6.99 | 98.44 | 3.98 |
|  | 5000 | 98.89 | 9.45 | 104.46 | 3.36 |
| ARTI | 20 | 98.51 | 7.17 | 98.77 | 8.27 |
|  | 1000 | 92.50 | 6.13 | 90.55 | 5.87 |
|  | 5000 | 104.89 | 7.59 | 104.98 | 2.23 |
| DHAA | 160 | 90.44 | 5.69 | 94.69 | 8.68 |
|  | 1000 | 90.28 | 7.89 | 94.23 | 3.92 |
|  | 5000 | 98.08 | 3.01 | 96.98 | 2.73 |
| AA | 160 | 102.49 | 6.39 | 98.35 | 1.49 |
|  | 1000 | 102.84 | 9.00 | 104.86 | 4.28 |
|  | 5000 | 106.24 | 5.49 | 103.09 | 4.90 |
