# Supplementary material for: The plant matrix of Artemisia annua L. for the treatment of malaria: Pharmacodynamic and pharmacokinetic studies
Source: PLoS One. 2025 May 7;20(5):e0322835. doi: 10.1371/journal.pone.0322835 (PMC12058161; doi:10.1371/journal.pone.0322835)
Supplement: S3 Table — (DOCX) [file pone.0322835.s009.docx]

**S3 Table Results of stability studies of five compounds in SD rat plasma using UHPLC-ESI-MS/MS (n=5)**

| Compound | Concentration (ng/mL) | Room temperature 24h | | | 3 freeze-thaw cycles | | | 7 days at -80°C | | |
| --- | --- | --- | --- | --- | --- | --- | --- | --- | --- | --- |
|  |  | mean ± SD | RSD (%) | Accuracy | mean ± SD | RSD (%) | Accuracy | mean ± SD | RSD (%) | Accuracy |
| ART | 40 | 41.1 ± 1.49 | 3.62% | 102.70% | 38.5 ± 2.35 | 6.10% | 96.14% | 40.3 ± 0.80 | 2.00% | 100.80% |
|  | 1000 | 1070 ± 40.9 | 3.82% | 106.95% | 1053 ± 29.9 | 2.84% | 105.28% | 1051 ± 29.5 | 2.80% | 105.14% |
|  | 5000 | 5229 ± 418 | 7.99% | 104.57% | 4629 ± 113 | 2.44% | 92.59% | 4673 ± 65.9 | 1.41% | 93.45% |
| DEART | 20 | 21.6 ± 0.78 | 3.59% | 108.22% | 21.4 ± 1.03 | 4.82% | 106.81% | 21.1 ± 1.73 | 8.16% | 105.75% |
|  | 1000 | 1093 ± 39.5 | 3.61% | 109.30% | 1069 ± 21.8 | 2.04% | 106.94% | 1069 ± 21.6 | 2.02% | 106.86% |
|  | 5000 | 5205 ± 494 | 9.49% | 104.10% | 4765 ± 110 | 2.31% | 95.30% | 4705 ± 267 | 5.68% | 94.10% |
| ARTI | 20 | 21.1 ± 0.78 | 3.67% | 105.62% | 21.0 ± 1.74 | 8.26% | 105.22% | 20.6 ± 1.03 | 4.98% | 103.01% |
|  | 1000 | 1003 ± 39.5 | 3.94% | 100.29% | 942 ± 21.5 | 2.28% | 94.19% | 920 ± 31.6 | 3.43% | 92.03% |
|  | 5000 | 4765 ± 218 | 4.58% | 95.29% | 4532 ± 63.0 | 1.39% | 90.64% | 4563 ± 143 | 3.14% | 91.25% |
| DHAA | 160 | 151 ± 5.81 | 3.85% | 94.40% | 161 ± 10.8 | 6.71% | 100.54% | 149 ± 5.80 | 3.89% | 93.23% |
|  | 1000 | 960 ± 45.4 | 4.73% | 95.97% | 914 ± 35.7 | 3.91% | 91.43% | 1064 ± 21.4 | 2.01% | 106.36% |
|  | 5000 | 5079 ± 336 | 6.62% | 101.58% | 4813 ± 108 | 2.24% | 96.27% | 4814 ± 118 | 2.45% | 96.28% |
| AA | 160 | 152 ± 6.95 | 4.58% | 94.94% | 175 ± 7.36 | 4.19% | 109.65% | 169 ± 8.62 | 5.10% | 105.52% |
|  | 1000 | 956 ± 68.0 | 7.12% | 95.58% | 948 ± 56.6 | 5.98% | 94.77% | 919 ± 34.8 | 3.78% | 91.94% |
|  | 5000 | 4689 ± 298 | 6.36% | 93.78% | 4777 ± 82.6 | 1.73% | 95.55% | 4738 ± 174 | 3.68% | 94.76% |
