# Supplementary material for: The plant matrix of Artemisia annua L. for the treatment of malaria: Pharmacodynamic and pharmacokinetic studies
Source: PLoS One. 2025 May 7;20(5):e0322835. doi: 10.1371/journal.pone.0322835 (PMC12058161; doi:10.1371/journal.pone.0322835)
Supplement: S1 File — (PDF) [file pone.0322835.s010.pdf]

^{1}H and ^{13}C NMR data of sesquiterpene components

All ^{1}H and ^{13}C NMR experiments were recorded on either a Bruker 500 MHz AVANCE II NMR Spectrometer. Chemical shifts are expressed in ppm (δ) relative to TMS as internal standard.

Arteannuin I (ARTI)

White needle crystal. NMR data for: 1H NMR (500 MHz,CDCl3) δ: 5.05 (d, *J*=1.5 Hz, 1H, H-15), 4.96 (d, *J*=11.9 Hz, 1H, H-5), 4.81 (d, *J*=1.7 Hz, 1H, H-15), 2.74-2.63 (m, 1H), 2.25 (ddd, *J*=13.8, 4.4, 2.2 Hz, H-3), 2.04-1.84(m, H-3), 1.81-1.70 (m, 2H, H-1), 1.57 (dd, *J*=25.7, 20.4 Hz, 1H), 1.47 (tt, *J*=13.8, 4.7 Hz, 1H), 1.37-1.24 (m, 1H), 1.24-1.21(m, 3H), 1.04 (ddd, *J*=25.3, 13.3, 3.9 Hz, 1H, H-9), 0.91(d, *J*=6.3 Hz, 3H, H-14); 13C NMR (126 MHz, CDCl3) δ: 43.84 (C-1), 28.83 (C-2), 29.67 (C-3), 146.28 (C-4), 45.64 (C-6), 40.13 (C-7), 22.82 (C-8), 35.12 (C-9), 28.24 (C-10), 40.6 (C-11), 174.54 (C-12), 13.48 (C-13), 20.2 (C-14), 105.26 (C-15) (S4 and S5 Figs).

Deoxyartemisinin (DEART)

White powdery crystal. NMR data for: ^{1}H NMR (500 MHz, CDCl3) δ: 5.69 (s, 1H, H-5), 3.27-3.10 (m, 1H), 2.01 (dt, *J*=12.9, 4.4 Hz, 1H, H-7), 1.96-1.87 (m, 2H), 1.84-1.74 (m, 2H), 1.68-1.56 (m, 1H), 1.53 (s, 3H, H-15), 1.33-1.21 (m, 3H), 1.20 (d, *J*=7.2 Hz, 3H, H-13), 1.10 (ddd, *J*=13.0, 12.1, 5.6 Hz, 1H), 1.00 (ddd, *J*=16.6, 13.0, 3.0 Hz, 1H), 0.94 (d, *J*=5.8 Hz, 3H, H-14); 13C NMR (126 MHz, CDCl3) δ: 44.74 (C-1), 22.15 (C-2), 34.1 (C-3), 109.35 (C-4), 99.78 (C-5), 82.54 (C-6), 42.54 (C-7), 23.65 (C-8), 33.58 (C-9), 35.49 (C-10), 32.89 (C-11), 172.00 (C-12), 12.76 (C-13), 18.72 (C-14), 24.11 (C-15) (S4 and S5 Figs).

Dihydroartemisinic acid (DHAA)

White powdery crystal. NMR data for: ^{1}H NMR (500 MHz, CDCl3) δ: 5.12 (s, 1H, H-5), 2.56-2.43 (m, 2H), 2.02-1.71 (m, 3H), 1.69-1.49 (m, 6H), 1.48-1.37 (m, 2H), 1.34-1.22 (m, 1H), 1.19 (d, *J*=6.9 Hz, 3H, H-13), 1.17-0.91 (m, 2H), 0.87 (d, *J*=6.5 Hz, 3H, H-14); ^{13}C NMR (126 MHz, CDCl3) δ: 43.88 (C-1), 26.06 (C-2), 26.90 (C-3), 136.31 (C-4), 119.58 (C-5), 36.63 (C-6), 42.39 (C-7), 27.71 (C-8), 35.52 (C-9), 27.95 (C-10), 42.00 (C-11), 183.39 (C-12), 15.38 (C-13), 19.99 (C-14), 24.12 (C-15) (S4 and S5 Figs).

Artemisinic acid (AA)

White powdery crystal. NMR data for: ^{1}H NMR (500 MHz, CDCl3) δ: 6.45 (s, 1H, H-13), 5.56 (s, 1H, H-13), 4.98 (s, 1H, H-5), 2.85-2.50 (m, 2H), 1.98-1.83 (m, 2H), 1.83-1.62 (m, 2H), 1.59 (s, 3H, H-15), 1.58-1.28 (m, 5H), 1.07 (qd, *J*=12.5, 4.0 Hz, 1H), 0.93-0.67 (m, 3H); ^{13}C NMR (126 MHz, CDCl3) δ: 41.38 (C-1), 25.55 (C-2), 26.38 (C-3), 134.97 (C-4), 120.14 (C-5), 37.88 (C-6), 42.05 (C-7), 25.93 (C-8), 35.22 (C-9), 27.57 (C-10), 142.55 (C-11), 172.34 (C-12), 126.65 (C-13), 19.75 (C-14), 23.71 (C-15) (S4 and S5 Figs).
